# The anti-cholesterolaemic effect of a consortium of probiotics: An acute study in C57BL/6J mice

**DOI:** 10.1038/s41598-017-02889-5

**Published:** 2017-06-06

**Authors:** D. R. Michael, T. S. Davies, J. W. E. Moss, D. Lama Calvente, D. P. Ramji, J. R. Marchesi, A. Pechlivanis, S. F. Plummer, T. R. Hughes

**Affiliations:** 1 Cultech Limited, Unit 2 Christchurch Road, Baglan Industrial Park, Port Talbot SA12 7BZ United Kingdom; 2 Cardiff School of Biosciences, Cardiff University, Sir Martin Evans Building, Museum Avenue, Cardiff CF14 3AX United Kingdom; 3 Centre for Digestive and Gut Health, Imperial College London, London, SW7 2AZ United Kingdom; 4 Division of Infection and Immunity, Henry Wellcome Building, Cardiff University, Cardiff, CF14 4XN United Kingdom; 5 Division of Computational and Systems Medicine, Department of Surgery and Cancer, Faculty of Medicine, Imperial College London, SW7 2AZ London, United Kingdom

## Abstract

Hypercholesterolaemia is a major risk factor for cardiovascular disease and it has been found that some probiotic bacteria possess cholesterol-lowering capabilities. In this study, the ability of the Lab4 probiotic consortium to hydrolyse bile salts, assimilate cholesterol and regulate cholesterol transport by polarised Caco-2 enterocytes was demonstrated. Furthermore, in wild-type C57BL/6J mice fed a high fat diet, 2-weeks supplementation with Lab4 probiotic consortium plus *Lactobacillus* *plantarum* CUL66 resulted in significant reductions in plasma total cholesterol levels and suppression of diet-induced weight gain. No changes in plasma levels of very low-density lipoprotein/low-density lipoprotein, high-density lipoprotein, triglycerides, cytokines or bile acids were observed. Increased amounts of total and unconjugated bile acids in the faeces of the probiotic-fed mice, together with modulation of hepatic small heterodimer partner and cholesterol-7α-hydroxylase mRNA expression, implicates bile salt hydrolase activity as a potential mechanism of action. In summary, this study demonstrates the cholesterol-lowering efficacy of short-term feeding of the Lab4 probiotic consortium plus *L. plantarum* CUL66 in wild-type mice and supports further assessment in human trials.

## Introduction

Cardiovascular disease (CVD) is the cause of death in one in three people in the United Kingdom^1^ and is the leading cause of global mortality^2^. Hypercholesterolaemia is a major risk factor for the disease and statins are widely used to normalise elevated circulating cholesterol levels and can reduce CVD-related events by approximately 25%^3–7^ and are often associated with adverse side effects^8^. Primary and secondary care of CVD imparts a heavy economic burden on society^1^. Management of modifiable lifestyle risk factors, such as diet, body weight and physical activity, represent preventative measures and are advocated by healthcare providers^7, 9^. However, the high mortality rates associated with CVD suggest these measures are not sufficiently effective and further options are required^10–12^.

Probiotics are defined as “live microorganisms that, when administered in adequate amounts, confer a health benefit on the host”^13, 14^ and there is growing evidence that some probiotic organisms possess a cholesterol lowering capability and could be considered as a potential supplemental tool in combatting CVD and associated conditions^15^. The cholesterol-lowering efficacy of a diversity of microbial species and strains has been observed^16–21^. There are multiple mechanisms by which these effects are thought to occur including the assimilation of cholesterol^22^ and/or the deconjugation of bile salts by bile salt hydrolase (BSH) positive probiotic bacteria that put increased demand on *de novo* bile synthesis (from circulating cholesterol) to replace that which is lost in faeces^23, 24^. Probiotic bacteria have also been shown to modulate key intestinal cholesterol transport pathways by regulating gene expression patterns of Niemann-Pick C1-like 1 (NPC1L1), ATP-binding cassette sub-family G member (ABCG)-5, ABCG-8 or ATP-binding cassette transporter-1 (ABCA-1) in intestinal epithelial cells^25–29^.

*L. plantarum* CUL66 (NCIMB 30280) has been found to have a cholesterol-lowering capability^29^ and the effects of the Lab4 consortium of probiotics (Lab4, composed of *Lactobacillus acidophilus* CUL21 (NCIMB 30156) and CUL60 (NCIMB 30157), *Bifidobacterium bifidum* CUL20 (NCIMB 30153) and *Bifidobacterium animalis* subsp*. lactis* CUL34 (NCIMB 30172) during other conditions are documented^30–33^. In the present study, assessment of the cholesterol lowering capabilities of Lab4 was made *in vitro* prior to its inclusion, in combination with *L. plantarum* CUL66, in a short-term feeding study with C57BL/6J mice on a high fat diet.

## Results

### Evidence for cholesterol lowering ability by Lab4 *in vitro*

**Figure 1:**
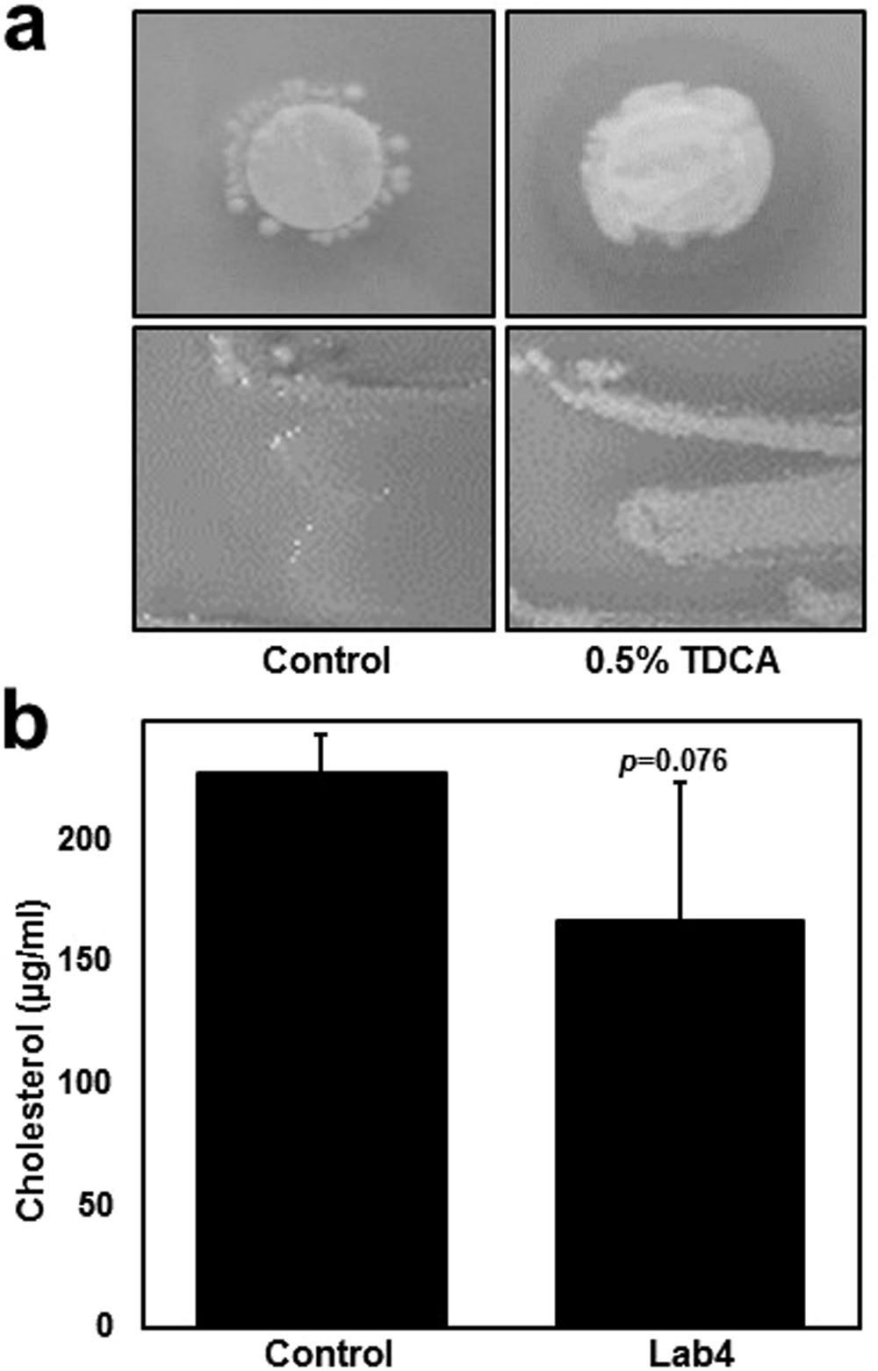
BSH activity and cholesterol assimilation by Lab4. (**a**) De Man, Rogosa and Sharpe (MRS) agar plates (control, top and bottom left-sided panels) or MRS agar plates containing 0.05% taurodeoxycholic acid (TDCA, top and bottom right-sided panels) that were inoculated with Lab4 on filter discs (top panels) or as bacterial streaks (bottom panels, n = 1) for 48 hours under anaerobic conditions. (**b**) Cholesterol concentration in MRS broth containing 0.3% (w/v) ox-bile and 200 µg/ml cholesterol (control) or in MRS broth containing 0.3% (w/v) ox-bile and 200 µg/ml cholesterol that were inoculated with Lab4 for 18 hours under anaerobic conditions. The data are presented as a representative image from 3 identical experiments (unless stated, Fig. 1a) or the mean ± SD from three independent experiments (Fig. 1b). Statistical analysis was performed using Student’s *t*-test and values of *p* are stated where appropriate.

BSH activity in Lab4 was indicated by the formation of a white precipitate and agar-clouding in the presence of 0.5% TDCA (Fig. 1a; right-sided panels) that was absent on control agar (Fig. 1a; left-sided panels). Growing cultures of Lab4 removed 26.54% (*p* = 0.076) of cholesterol from MRS broth (Fig. 1b) equating to 4.84 ± 5.27 mg of cholesterol per gram of dry weight bacteria.

**Figure 2:**
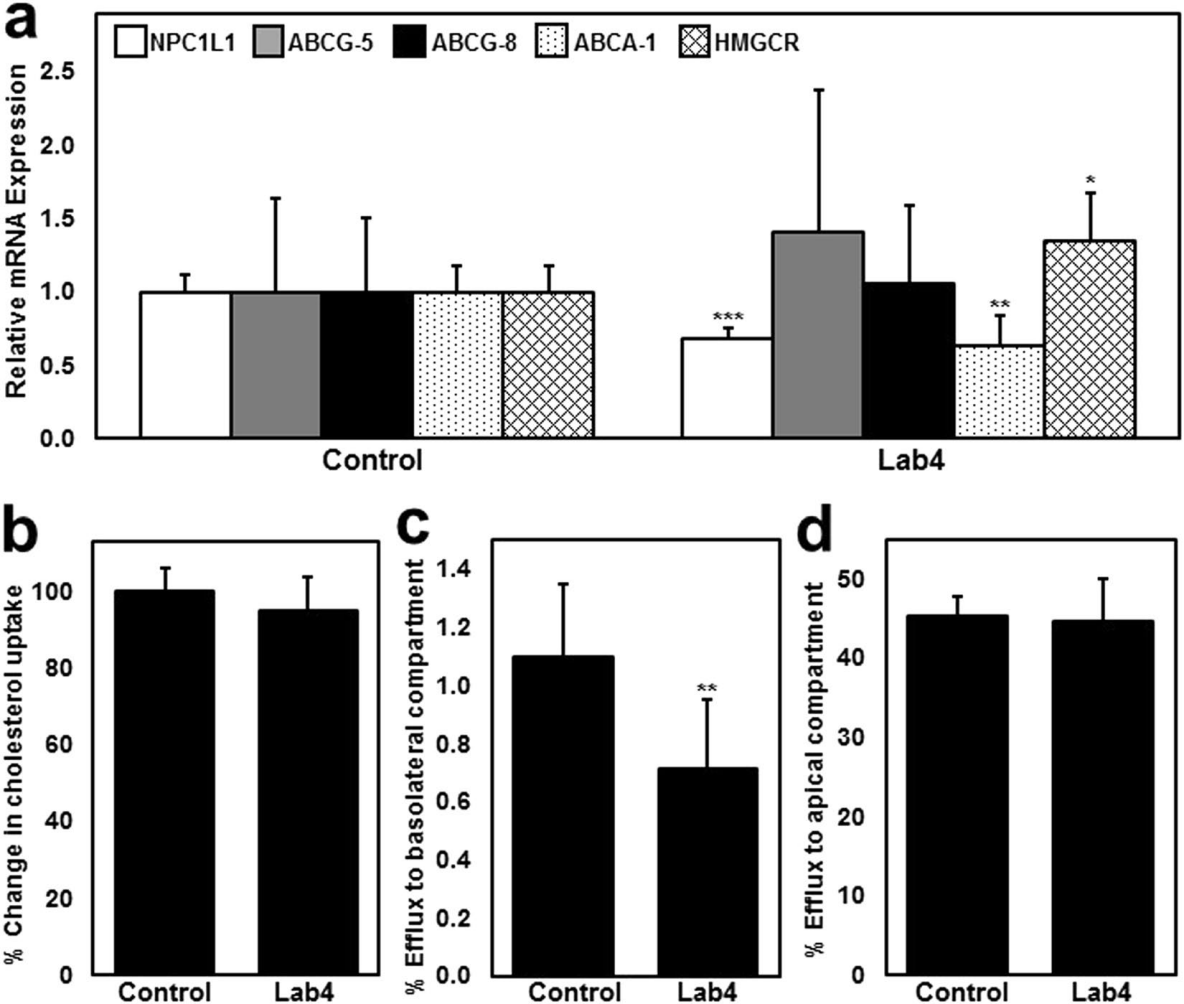
The effect of Lab4 on cholesterol homeostasis in Caco-2 enterocytes. (**a**) Gene transcript levels of NPC1L1, ABCG-5, ABCG-8, ABCA-1 and HMGCR in Caco-2 cells that were treated with 70 µg/ml cholesterol (Control) or cholesterol (70 µg/ml) and Lab4 (1 × 10^8^ cfu/ml) for 6 hours. Gene transcript levels were calculated using the comparative cycle threshold (C*t*) method and normalised to β-actin levels with the control given an arbitrary value of 1.0. (**b**) Cholesterol uptake by untreated (control) Caco-2 cells or those incubated with Lab4 (1 × 10^8^ cfu/ml) for 5 hours prior to the addition of radiolabelled cholesterol for an additional hour. Intracellular radioactivity (disintegrations per minute) was normalised to total protein content and presented as a percentage of the control that has been arbitrarily assigned as 100%. Efflux of intracellular radiolabelled cholesterol to apolipoprotein-AI (Apo-AI, 10 μg/ml) in the basolateral compartment (**c**) or TDCA micelles (1 nM) in the apical compartment (**d**) by untreated (control) Caco-2 cells or those treated with Lab4 (1 × 10^8^ cfu/ml) for 6 hours. The percentage of intracellular cholesterol effluxed from the cells was determined by dividing the radioactivity of the apical media or basolateral media by the combined radioactivity in the apical media, basolateral media and cell fraction. The data are presented as the mean ± SD from at least three independent experiments. Statistical analysis was performed using Student’s *t*-test where *p < 0.05, **p < 0.01 and ***p < 0.001.

As seen in Fig. 2a, when incubated with 21-day polarised Caco-2 cells and cholesterol (70 µg/ml), live cultures of Lab4 reduced NPC1L1 and ABCA-1 gene expression by 33% (*p* = 0.00002) and 37% (*p* = 0.001) respectively and increased 3-hydroxy-3-methylglutaryl-CoA reductase (HMGCR) gene expression by 35% (*p* = 0.012) when compared to cells treated with cholesterol alone (Control). No significant changes in ABCG-5 or ABCG-8 gene expression were observed. The viability of both Caco-2 cells and Lab4 was maintained throughout the experiment (Supplementary Figure S1).

No substantial changes in the uptake of extracellular radiolabelled cholesterol were observed in response to Lab4 (Fig. 2b) despite reduced expression of NPC1L1 (Fig. 2a) and the ability of Lab4 to assimilate cholesterol (Fig. 1b). Reduced gene expression of ABCA-1 (Fig. 2a) was also observed in response to Lab4 suggesting a possible impact on cholesterol transport. As seen in Fig. 2c, the apical application of viable Lab4 cultures to Caco-2 cells housed in a dual compartment trans-well system significantly reduced the basolateral efflux of intracellular radiolabelled cholesterol compared to the control (35%, *p* = 0.004). The magnitude of this reduction is in line with the 37% reduction in ABCA-1 gene expression (Fig. 2a). No changes in the efflux of intracellular radiolabelled cholesterol into the extracellular apical compartment were observed (Fig. 2d).

### Short-term feeding of mice with Lab4 and *L. plantarum* CUL66 reduces plasma total cholesterol (TC) and diet-induced weight gain but has no effect on plasma very low-density lipoprotein (VLDL)/low-density lipoprotein (LDL), high-density lipoprotein (HDL), triglycerides (TG) or cytokines

**Figure 3:**
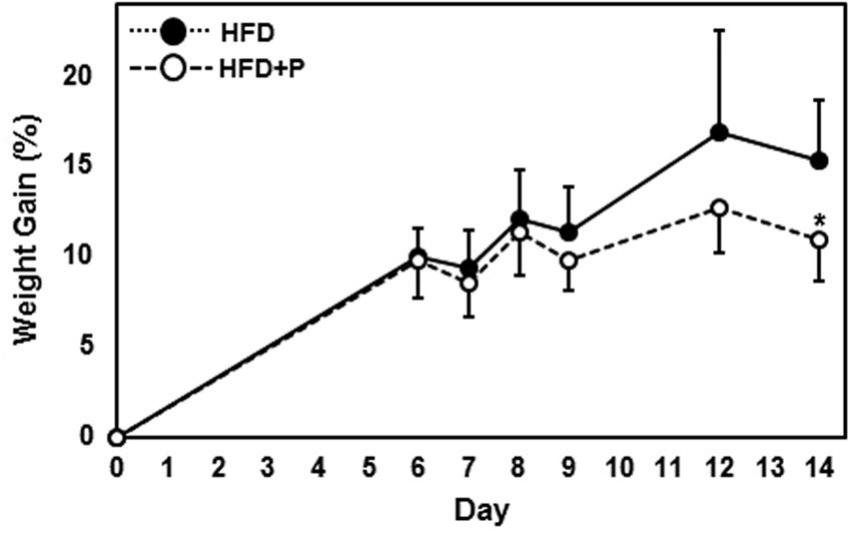
Effect of probiotics on body weight. Body weights of mice in the HFD and HFD + P groups were recorded throughout the intervention period at the indicated time points and the percentage change in body weight since day 0 was calculated for each mouse. Data is presented as the mean ± SD for 6 mice in each group. Statistical analysis was performed using Student’s *t*-test where *p < 0.05.

As expected, mice in the high fat diet (HFD) group presented elevated plasma levels of TC (2.89 mM vs 3.64 mM, *p* = 0.001), VLDL/LDL (0.41 mM vs 0.79 mM, *p* = 0.000003) and HDL (1.73 mM vs 2.04 mM, *p* = 0.054) compared to baseline (BL) values although TG levels remained unchanged (Table 1). Between group comparison showed that plasma TC was significantly reduced by 14% (3.64 mM vs 3.15 mM, *p* = 0.029) in the high fat diet plus probiotic (HFD + P) group compared to the HFD group to a level similar to BL levels. Plasma cytokines were not changed in response to the high fat diet (BL vs HFD) or probiotic feeding (HFD vs HFD + P) although significantly elevated levels of keratinocyte chemoattractant/growth-regulated oncogene were observed in the HFD + P group compared to BL levels (63.19 pg/ml vs 89.62 pg/ml, 42%, *p* = 0.045). Mice in the HFD + P group showed significantly less weight gain after 14 days feeding compared to the HFD group (10.92% vs 15.33% respectively, *p* = 0.025, Fig. 3). No residual food was found during daily cage checks.

**Table 1:** Plasma lipid and cytokine concentrations.

	BL	HFD	HFD + P
**Plasma Lipids (mM)**			
Total cholesterol	2.89 ± 0.09	3.64 ± 0.33**	3.15 ± 0.38^#^
Very low-density lipoprotein/low-density lipoprotein	0.41 ± 0.10	0.79 ± 0.07***	0.69 ± 0.09***
High-density lipoprotein	1.73 ± 0.14	2.04 ± 0.23^*p*=0.054^	1.79 ± 0.26
Triglycerides	0.76 ± 0.14	0.80 ± 0.15	0.71 ± 0.12
**Plasma Cytokines (pg/ml)**			
Interferon-γ	0.59 ± 0.26	0.61 ± 0.39	0.97 ± 0.41
Interleukin-10	14.23 ± 4.26	12.83 ± 2.56	15.18 ± 3.83
Interleukin-12p70	51.11 ± 54.01	30.05 ± 32.06	19.80 ± 12.17
Interleukin-1β	0.84 ± 0.59	0.74 ± 0.49	0.60 ± 0.24
Interleukin-2	3.24 ± 1.46	2.84 ± 1.41	2.14 ± 0.60
Interleukin-4	0.72 ± 0.57	0.43 ± 0.37	0.37 ± 0.13
Interleukin-5	2.95 ± 0.64	3.14 ± 1.20	3.18 ± 0.34
Interleukin-6	6.37 ± 2.13	10.22 ± 8.47	10.39 ± 3.80
Keratinocyte chemoattractant/growth-regulated oncogene	63.19 ± 15.62	75.36 ± 14.15	89.62 ± 21.26*
Tumor Necrosis Factor-α	10.7 1 ± 1.27	11.47 ± 2.59	15.34 ± 5.16

Data represent the means ± standard deviation of 6 mice per group. Values of *p* were determined using one-way ANOVA with Tukey’s (equal variance) or Dunnett’s T3 (unequal variance) post-hoc analysis where *∗* *p* < 0.05, *∗∗* *p* < 0.01, and *∗∗∗* *p* < 0.001 versus the BL group; ^#^ *p* < 0.05 versus the HFD group. Values of *p* compared to the BL group are stated where appropriate.

### Short-term feeding with Lab4 plus *L. plantarum* CUL66 has no effect on plasma bile acids, but increases faecal bile acid excretion

**Figure 4:**
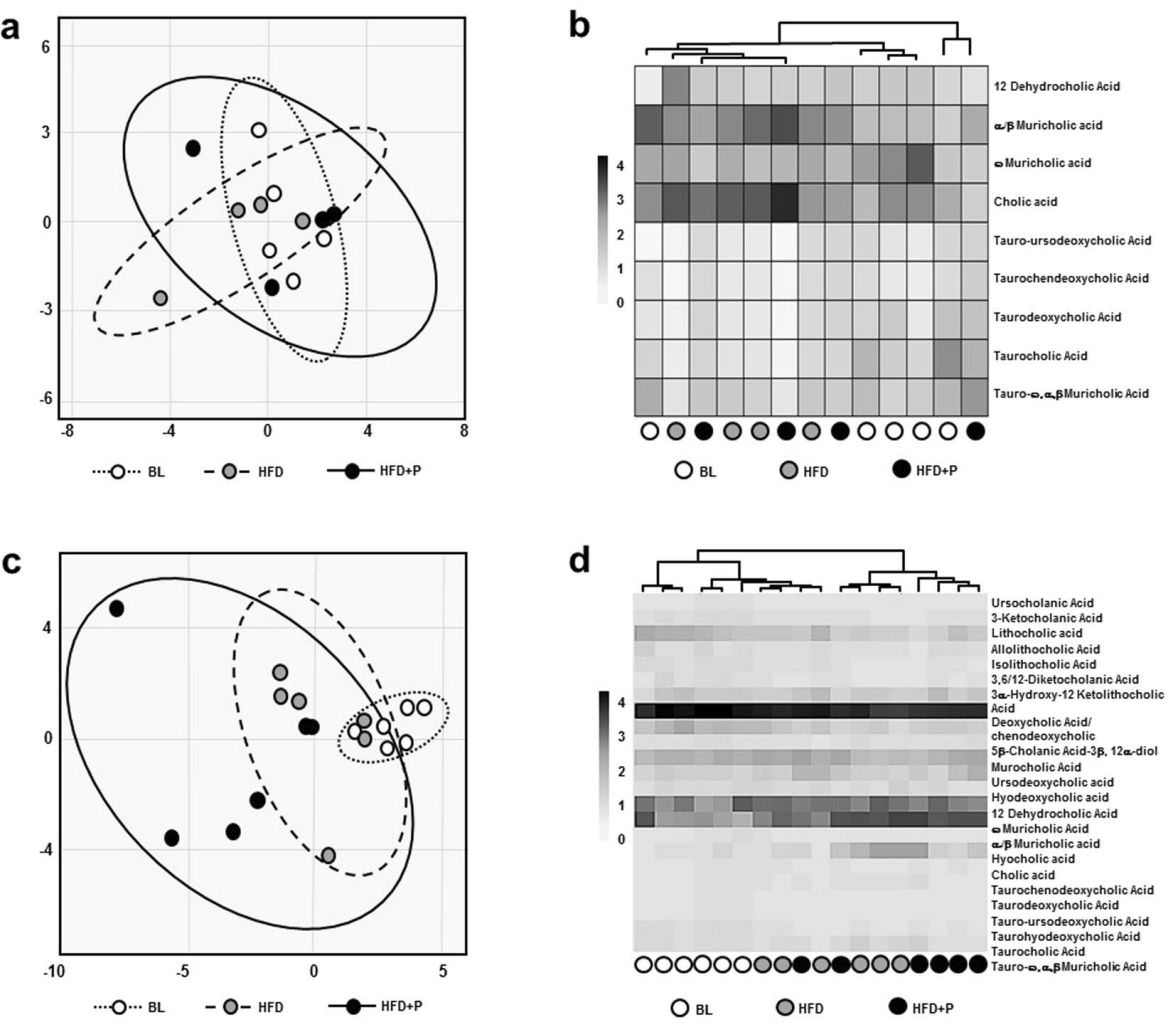
Principle component analysis (PCA) and heatmap analysis of plasma and faecal bile acid profiles. PCA score plots of bile acid signatures from the (**a**) plasma or (**c**) faeces of BL, HFD and HFD + P mice. Heatmaps of the bile acid relative intensity from (**b**) plasma or (**d**) faeces of each mouse.

Analysis of bile acids did not identify any differences between groups in the levels present in the plasma (Fig. 4a and b). In contrast, mice fed HFD and HFD + P have different faecal bile acid profiles to the BL group (Fig. 4c and d) and the faecal bile acid profile of HFD + P fed mice is more variable than those fed HFD alone (Fig. 4c). Plasma and faecal bile acid profiles (as relative intensities from UPLC-MS) are shown in Supplementary Table S1 and Supplementary Figure S2. Unlike plasma levels that remained unchanged, total and unconjugated bile acid levels were significantly increased in the faeces of both groups during the study compared to BL and significantly higher levels of total (34% increase, *p* = 0.047) and unconjugated bile acids (33% increase, *p* = 0.047) were present in the faeces of HFD + P mice compared to the HFD group (Table 2). Increased relative levels of ursodeoxycholic acid (46% increase, *p* = 0.003), hyodeoxycholic acid (96% increase, *p* = 0.028), taurochenodeoxycholic acid (180% increase, *p* = 0.026) and a trend towards an increase in deoxycholic/chenodeoxycholic acid (30%, *p* = 0.052) were observed in the faeces of HFD + P mice compared to the HFD group (Table 2).

**Table 2:** Ratio of plasma and faecal bile acid content in relation to BL group.

	PLASMA		FAECES	
	HFD	HFD + P	HFD	HFD + P
Total bile acids	1.39 ± 0.56	1.07 ± 1.20	1.72 ± 0.34*	2.30 ± 0.49***^#^
Conjugated bile acids	1.56 ± 1.13	0.90 ± 0.57	2.07 ± 1.21	3.62 ± 2.96*
Unconjugated bile acids	1.35 ± 0.48	1.11 ± 1.46	1.71 ± 0.33*	2.27 ± 0.46***^#^
**Individual bile acids**				
Ursocholanic Acid	ND	ND	1.16 ± 0.44	1.32 ± 0.47
3-Ketocholanic Acid	ND	ND	1.12 ± 0.51	1.39 ± 0.70
Lithocholic acid	ND	ND	1.11 ± 0.33	1.25 ± 0.46
Allolithocholic Acid	ND	ND	1.38 ± 0.65	1.98 ± 1.08
Isolithocholic Acid	ND	ND	1.11 ± 0.55	1.22 ± 0.55
3,6/12-Diketocholanic Acid	ND	ND	0.94 ± 0.80	1.58 ± 1.04
3α-Hydroxy-12 Ketolithocholic Acid	ND	ND	1.74 ± 0.61	2.47 ± 0.87*
Deoxycholic Acid/Chenodeoxycholic	ND	ND	1.29 ± 0.17	1.68 ± 0.28*^p=0.052^
5β-Cholanic Acid-3β, 12α-diol	ND	ND	0.94 ± 0.23	1.17 ± 0.37
Murocholic Acid	ND	ND	3.07 ± 1.77**	4.83 ± 2.54***
Ursodeoxycholic acid	ND	ND	1.58 ± 0.11*	2.31 ± 0.36**^##^
Hyodeoxycholic acid	ND	ND	2.26 ± 0.91**	4.42 ± 1.91***^#^
12-Dehydrocholic Acid	0.59 ± 0.27	0.59 ± 0.78	2.41 ± 1.09*	2.58 ± 1.29*
ω-Muricholic Acid	0.93 ± 0.32	0.59 ± 0.77	1.87 ± 0.39**	2.22 ± 0.46**
α/β-Muricholic acid	2.41 ± 0.99	1.94 ± 2.49	2.98 ± 1.19***	4.28 ± 1.29***
Hyocholic acid	ND	ND	1.20 ± 0.38	1.67 ± 0.43
Cholic acid	1.49 ± 0.63	1.35 ± 1.82	17.00 ± 16.70	18.82 ± 15.33***
Taurochenodeoxycholic Acid	9.18 ± 9.09*	4.36 ± 3.04	2.99 ± 1.32*	8.40 ± 5.75***^#^
Taurodeoxycholic Acid	1.08 ± 0.88	0.86 ± 0.53	6.12 ± 4.21**	13.75 ± 13.81***
Tauro-ursodeoxycholic Acid	1.50 ± 0.26	1.16 ± 0.66	3.88 ± 2.51*	6.25 ± 4.50**
Taurohyodeoxycholic Acid	ND	ND	2.73 ± 1.50*	7.25 ± 5.46**
Taurocholic Acid	0.88 ± 0.82	0.50 ± 0.43	0.60 ± 0.22	1.29 ± 1.08
Tauro-ω,α,β Muricholic Acid	1.99 ± 1.27	1.04 ± 0.62	2.70 ± 1.76	3.64 ± 2.64**

Data represent the means ± SD of 4 (plasma) or 6 (faeces) mice per group. Values of *p* were determined using one-way ANOVA with Tukey’s (equal variance) or Dunnett’s T3 (unequal variance) post-hoc analysis where *∗* *p* < 0.05, *∗∗* *p* < 0.01, and *∗∗∗* *p* < 0.001 versus the BL group; ^#^ *p* < 0.05, ^##^ *p* < 0.01, and ^###^ *p* < 0.001 versus the HFD group. Values of *p* compared to the HFD group are stated where appropriate. ND, not detected.

### Short-term feeding with Lab4 plus *L. plantarum* CUL66 modulates the expression of genes associated with hepatic bile metabolism, but has no effect on those associated with cholesterol metabolism/transport in the duodenum, colon or liver

Significantly reduced mRNA levels of small heterodimer partner (SHP, 42%, *p* = 0.010) and significantly elevated mRNA levels of cholesterol-7α-hydroxylase **(**CYP7A1, 84%, *p* = 0.047) were observed in the livers of the HFD + P mice when compared to those fed HFD alone (Table 3). A trend towards an increase in hepatic ABCG-8 mRNA (23%, *p* = 0.067) was observed in HFD + P when compared to those fed HFD alone (Table 3). No significant differences between groups in the expression of farnesoid X receptor (FXR), NPC1L1, ABCG-5, ABCG-8, ABCA-1 and HMGCR were observed in duodenal, colonic or liver tissues (Table 3). Fibroblast growth factor 15 (FGF15) mRNA could not be detected in the duodenum or colon (Table 3).

**Table 3:** Ratio of expression of key genes involved in bile acid and cholesterol metabolism in the HFD+P group in relation to the HFD group.

	DUODENUM	COLON	LIVER
FXR	1.26 ± 1.20	1.78 ± 1.09	1.27 ± 0.25
FGF15	ND	ND	NT
SHP	NT	NT	0.58 ± 0.18*
CYP7A1	NT	NT	1.84 ± 0.69*
HMGCR	0.87 ± 0.72	2.16 ± 1.56	0.95 ± 0.19
NPC1L1	0.91 ± 0.56	0.89 ± 0.61	1.37 ± 0.35
ABCG5	1.14 ± 0.31	0.92 ± 0.68	1.03 ± 0.17
ABCG8	1.00 ± 1.20	0.78 ± 0.47	1.23 ± 0.20^*p*=0.067^
ABCA1	0.60 ± 0.66	0.75 ± 0.55	0.96 ± 0.31

Data represent the means ± SD of 6 mice per group values of *p* were determined using Student’s *t*-test where *∗* *p* < 0.05. Values of *p* compared to the HFD group are stated where appropriate. NT, not tested; ND, not detected.

## Discussion

In this short-term feeding study, daily probiotic supplementation resulted in lower plasma cholesterol levels and suppression of diet-induced weight gain in mice fed a high fat diet. High circulating levels of TC and obesity are associated with increased risk of CVD and reductions in cholesterol levels and body weight can have a beneficial impact on this disease^2^. The cholesterol-lowering and anti-obesity effects observed for Lab4 plus *L. plantarum* CUL66 support the findings of longer term feeding studies in C57BL/6J mice with other probiotics^34–40^ and further implicate probiotic supplementation as a potential strategy for the prevention of metabolic disease.

The deconjugation of bile acids by bacterial BSH activity is considered a key probiotic cholesterol-lowering mechanism^22, 26, 41–44^ and can increase faecal bile acid excretion in C57BL/6J mice^35, 45^ by repressing the enterohepatic FXR-FGF15 axis^45^ and increasing hepatic bile acid synthesis^35, 45^. In this study, we propose that reduced plasma cholesterol levels (Table 1) are the consequence of increased bile synthesis *de novo* by the host in response to probiotic-mediated bile acid deconjugation in the intestines. This view is supported by numerous obsevations: firstly, the ability of Lab4 (Fig. 1a) and *L. plantarum* CUL66^29^ to deconjugate bile acids *in vitro*. Secondly, increased levels of total and unconjugated bile acids in the faeces of probiotic fed mice (Table 2). Thirdly, a reduction in hepatic gene expression of SHP; a transcriptional repressor of CYP7A1; the rate limiting enzyme in the synthesis of bile salts from cholesterol^46^. Finally, the concomitant increase in hepatic mRNA CYP7A1 levels (Table 3) that has consistently shown direct correlation with increased hepatic CYP7A1 protein levels in numerous other studies^47–49^. It should also be noted that these changes were not accompanied with changes in plasma bile acids (Table 2) or intestinal FXR mRNA expression (Table 3) in accordance with observations made elsewhere^45^, although gene expression levels in the ileum; the key site of FXR-FGF15 activation^50, 51^, were not assessed in our study due to non-availability of tissue.

Probiotics have also been shown to lower cholesterol levels by regulating cholesterol transport and metabolism^25–27, 29^ and both Lab4 (Fig. 2a) and *L. plantarum* CUL66^29^ have been shown to inhibit the expression of the cholesterol uptake transporter NPC1L1 in human intestinal epithelial cells. For *L. plantarum* CUL66, decreased expression was associated with decreased extracellular cholesterol uptake together with increased expression of apical cholesterol efflux proteins ABCG-5 and ABCG-8^29^, but was not associated with any changes in cholesterol export into the apical compartment^29^. Lab4 and *L. plantarum* CUL66 have been shown to reduce the expression of the cholesterol efflux transporter ABCA-1 which may be linked to the observed reductions of ApoAI-mediated basolateral cholesterol efflux (Fig. 2c)^29^. Interestingly, transcript levels of the *de novo* cholesterol synthesis enzyme HMGCR were increased and this change could represent a compensatory mechanism used to maintain cellular cholesterol levels^29, 52–54^. The changes in gene expression that had been observed in cultured human Caco-2 enterocytes *in vitro* may represent a species-specific effect as similar changes were not detected in the duodenal or colonic tissue analysed on completion of the murine feeding study (Table 3).

Assimilation of cholesterol is thought to be another mechanism by which probiotic bacteria can influence plasma cholesterol levels^55^ although the impact of cholesterol assimilation by Lab4 (Fig. 1b) and *L. plantarum* ^29^ seen *in vitro* could not be assessed *in vivo* due a limited availability of faecal sample. Likewise, it was not possible to assess the impact of other potential probiotic cholesterol-lowering mechanisms such as the conversion of cholesterol to coprostanol, short chain fatty acid production^55^ and the assimilation of bile acids^56, 57^. Probiotics have also been shown to reduce blood TG levels^37, 38^ or impart anti-inflammatory effects^34, 40^ in HFD fed C57BL/6J mice but these responses were not seen in our study (Table 1) possibly as a result of the short intervention period.

In summary, this preliminary 2 week study in mice on a high fat diet demonstrated the cholesterol-lowering capability of a combination of Lab4 and *L. plantarum* CUL66 probiotic bacteria. The probiotic group presented lower plasma TC levels and reduced weight gain together with changes in faecal bile acid content which implicates the deconjugation of bile salts as a potential mechanism of action. These findings provide a meaningful basis for the design of follow-up studies to assess cholesterol lowering efficacy of these probiotic bacteria in humans.

## Methods

### Reagents

All chemicals were purchased from Sigma-Aldrich (Poole, UK) unless otherwise stated.

### Studies *in vitro*

Lab4 was assessed for its ability to deconjugate bile salts, assimilate cholesterol and regulate Caco-2 cell cholesterol transport as previously described^29^. Overnight cultures of Lab4 grown in MRS broth (Oxoid, Hampshire, UK) were centrifuged (1,000 × *g*, 10 min), washed with phosphate buffered saline and re-suspended to 1 × 10^8^ cfu/ml in antibiotic free Dulbecco’s Modified Eagle’s medium supplemented with 4.5 g/l glucose, 1% (v/v) non-essential amino acids and 10 mM HEPES.

### Studies *in vivo*

#### Maintenance of animals and feeding of probiotics

Eighteen 8-week-old male C57BL/6 J mice were obtained from Harlan Laboratories (Oxfordshire, UK) and housed in pathogen-free scantainer ventilated cages (3 mice per cage) in a light- and temperature-controlled facility (lights on 7 a.m. −7 p.m., 22 °C). After one week acclimatisation on standard chow diet, 6 mice were sacrificed for baseline analysis and the remaining 12 mice were randomly assigned into 2 groups; one group on high fat diet containing 21% (w/w) pork lard supplemented with 0.15% (w/w) cholesterol (Special Diets Services, Witham, U.K; product code: 821424) and the other on high fat diet supplemented with 5 × 10^8^ cfu/mouse/day of Lab4 plus *L. plantarum* CUL66. The mice were fed 10 g/cage/day (44.50 kcal/cage/day) for 14 days and cages were checked daily for surplus food. Body weights were recorded throughout the feeding period and faecal samples collected at the beginning and end of the study and were stored under anaerobic conditions at −80 °C for further analysis. At the end of the feeding period, all mice were terminally exsanguinated under anaesthesia by cardiac puncture and death confirmed by cervical dislocation. All studies and protocols were approved by the Cardiff University institutional ethics review committee and the United Kingdom Home Office and experiments were performed in accordance with the Guide for the Care and Use of Laboratory Animals (NIH Publication No. 85–23, revised 1996).

#### Blood and tissue sampling

Blood obtained from cardiac puncture was collected into EDTA (10 mM final concentration) and the plasma separated by centrifugation (12,000 × *g*, 5 mins). Liver and intestinal tissue was snap frozen in liquid nitrogen. All samples were stored at −80 °C.

#### Plasma lipid and cytokine analysis

TC, HDL and TG concentrations were measured at the Clinical Biochemistry Service, Cardiff University, on an Aeroset automated analyzer (Abbott Diagnostics, Berkshire, UK). Plasma VLDL/LDL concentrations were measured using the VLDL/LDL cholesterol assay kit (ABCAM, Cambridge, UK) in accordance with the manufacturer’s instructions. Plasma cytokine concentrations were measured by the Central Biotechnology Service (Cardiff University, UK) using the VPLEX pro-inflammatory panel 1 mouse kit (Meso Scale Discovery, Maryland, USA).

#### UPLC-MS profiling of plasma and faecal bile acids

Plasma samples were prepared for analysis using a previously described method^58^. Faecal pellets were lyophilised for 48 hours using a VirTis Benchtop BTP 8ZL freeze dryer (BPS FUK). The dried pellets were weighed and then homogenised in a mixture of water, acetonitrile and 2-propanol (2:1:1 vol.) using a Biospec bead beater with 1.0 mm Zirconia beads to extract bile acids. After centrifugation (16,000 × *g*, 20 mins) the supernatant was passed through 0.45 μm microcentrifuge filters (Costar, Corning). Equal parts of the plasma and faecal filtrates were used for the preparation of quality control (QC) samples that are required to monitor the stability of the assay. QC samples were also spiked with mixtures of bile acid standards (55 bile acid standards including 36 non-conjugated, 12 conjugated with taurine, 7 conjugated with glycine (Steraloids, Newport, RI)) to determine the chromatographic retention times of bile acids. The filtrate was transferred in LCMS vials and used for the subsequent analysis. Plasma and faecal bile acid analysis was performed by ACQUITY UPLC (Waters Ltd, Elstree, UK) coupled to a Xevo G2 Q-ToF mass spectrometer equipped with an electrospray ionization source operating in negative ion mode (ESI-), using the method described by Sarafian *et al*.^58^. Waters raw data files were converted to NetCDF format and data were extracted via XCMS (v1.50) package with R (v3.1.1) software. MassLynx software 4.1 was used respectively for data acquisition and validation for this high throughput semi-targeted method for relative quantification of bile acids. Relative faecal bile acid intensities were corrected to the faecal pellet dry weight. PCA was carried out on the integrated mass spectrometric data using pareto scaling and logarithmic transformation using SIMCA v14.1.0.2047 (MKS Umetrics, Umeå, Sweden). The heatmaps were generated in R using package NMF using the scale command for the columns to create Z-scores.

#### Gene expression analysis

Approximately 50 mg of liver or intestinal scrapings were transferred into prefilled Lysis Matrix D micro-centrifuge tubes (MP Biomedicals, UK) containing 500 µl of Ribozol (Amresco LLC, Ohio, USA). The tissue was homogenized for 60 seconds at 6.0 m/s on a Fastprep-24^TM^ homogenizer (MP Biomedicals, UK) and total RNA was isolated in accordance with the manufacturer’s instructions (Ribozol, Amresco LLC, Ohio, USA). Total RNA (1 µg) was reverse transcribed into cDNA using the High Capacity cDNA reverse transcription Kit (Life Technologies, Paisley, UK) in accordance with the manufacturer’s instructions and real-time quantitative polymerase chain reaction (RT-qPCR) was performed on 10 ng cDNA using the iTag Universal SYBR Green SuperMix (Bio-Rad, Hertfordshire, UK) and 50 nM of each gene-specific oligonucleotide primer (See Supplementary Table S2): Initial melting (95 °C for 5 minutes) followed by 40 cycles of melting (94 °C for 15 seconds), annealing (60 °C for 15 seconds) and extension (72 °C for 30 seconds) was performed using a CFX Connect^TM^ Real-Time Instrument (Bio-Rad). Transcript levels in the HFD + P group were determined using 2^−(ΔC*t*1–ΔC*t*2)^, where ΔC*t* represents the difference between the C*t* for each target gene and β-Actin mRNA transcript levels and are expressed as a ratio of expression relative to the HFD group.

### Statistical analysis

All data are presented as the mean ± standard deviation (SD) of the assigned number of independent experiments or number of mice. Prior to significance testing, the normality of the data and the equality of group variance were confirmed using the Shapiro-Wilk and Levene’s tests respectively. Where necessary, normality was achieved using logarithmic transformation. For single comparisons, values of *p* were determined using Student’s *t*-test. For multiple comparisons, values of *p* were determined using one-way analysis of variance (ANOVA) with Tukey’s (equal variance) or Dunnett’s T3 (unequal variance) post-hoc analysis. Statistical analysis was performed using SPSS statistical software package version 22 (IBM, New York, USA). Significance was defined when *p* < 0.05.

### Data availability statement

The datasets generated during and/or analysed during the current study are available from the corresponding author on reasonable request.

## Electronic supplementary material

Supplementary information
