# Supplementary material for: The anti-cholesterolaemic effect of a consortium of probiotics: An acute study in C57BL/6J mice
Source: Sci Rep. 2017 Jun 6;7:2883. doi: 10.1038/s41598-017-02889-5 (PMC5460276; doi:10.1038/s41598-017-02889-5)
Supplement: Supplementary file 1 — Supplementary information [file 41598_2017_2889_MOESM1_ESM.pdf]

The anti-cholesterolemic effect of a consortium of probiotics: An acute study in C57BL/6 mice.

D. R. Michael, T. S. Davies, J. W. E. Moss, D. Lama Calvente, D. P. Ramji, J. R. Marchesi, A. Pechlivanis, S. F. Plummer and T. R. Hughes.

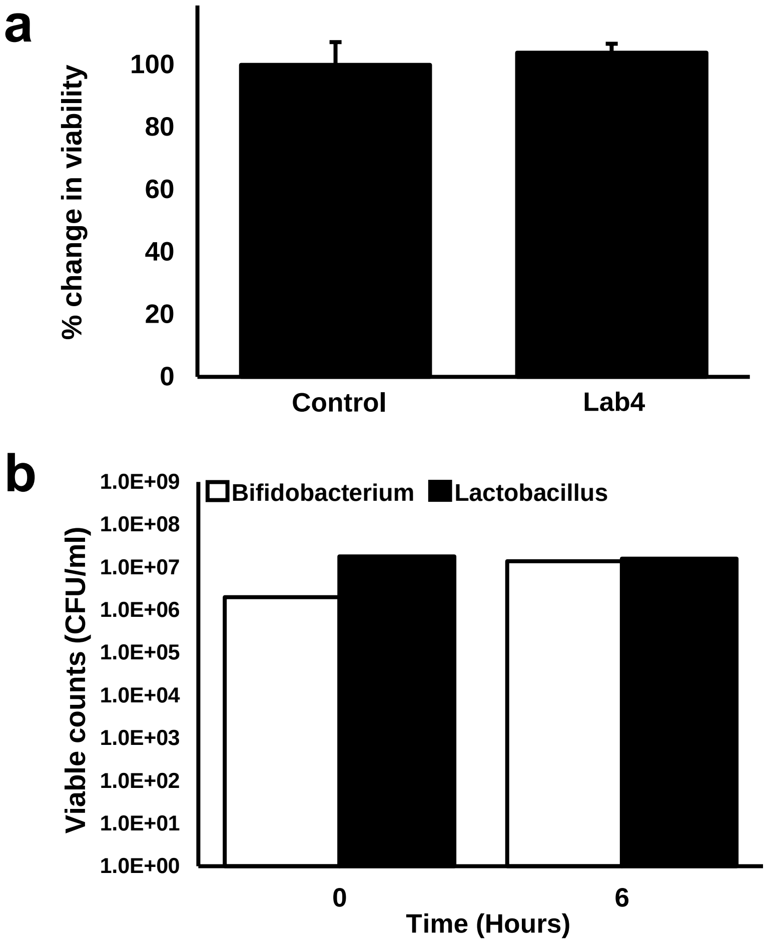

Supplementary Figure S1: Caco-2 cell and bacterial viability under experimental conditions. (*a*) 21 day polarized Caco-2 cells were treated with cholesterol (70 µg/ml; Control) or treated with cholesterol (70 µg/ml) and Lab4 ($1x10^8$ cfu/ml) for six hours (37°C, 5% CO_2). Cell viability was assessed using crystal violet staining as described elsewhere[1] and is expressed as a percentage of the control that has been assigned as 100%. The data are presented as the mean ± SD of triplicate samples from a single experiment. (*b*) Overnight cultures of Lab4 were adjusted to $1x10^8$ cfu/ml in supplemented antibiotic free DMEM and incubated with 21 day polarized Caco-2 cells and cholesterol (70 µg/ml) for six hours (37°C, 5% CO_2). Viable numbers of lactobacillus and Bifidobacterium were enumerated at the start and end of the incubation period on MRS agar or MRS agar supplemented with cysteine, propionate and lithium chloride respectively. The data are present as cfu/ml and represent a single experiment.

	PLASMA			FAECES		
	BL	HFD	HFD+P	BL	HFD	HFD+P
Ursocholanic Acid	ND	ND	ND	651.11±309.45	752.25±288.97	859.79±308.52
3-Ketocholanic Acid	ND	ND	ND	6218.40±1800.47	6978.47±3168.08	8647.75±4371.63
Lithocholic acid	ND	ND	ND	55686.70±6982.86	61941.92±18389.11	69574.73±25591.57
Allolithocholic Acid	ND	ND	ND	7760.32±5769.40	10683.85±5014.84	15342.52±8354.30
Isolithocholic Acid	ND	ND	ND	8319.61±2248.66	9205.53±4547.49	10142.26±4536.08
3,6/12-Diketocholanic Acid	ND	ND	ND	6090.77±4295.23	5710.73±4857.41	9639.33±6317.06
3α-Hydroxy-12 Ketolithocholic Acid	ND	ND	ND	31961.08±12472.75	55636.22±19554.80	78913.29±27841.08
Deoxycholic Acid/ chenodeoxycholic	ND	ND	ND	351927.23±133335.86	454251.81±59228.63	591466.30±99855.36
5β-Cholanic Acid-3β, 12α-diol	ND	ND	ND	58092.39±18262.26	54859.71±13132.50	67977.99±21774.81
Murocholic Acid	ND	ND	ND	3551.89±1412.48	10914.88±6280.14	17170.64±9017.83
Ursodeoxycholic acid	ND	ND	ND	66705.48±24069.68	105585.77±7365.31	154035.97±23833.54
Hyodeoxycholic acid	ND	ND	ND	22028.25±6293.33	49789.98±20052.01	97425.51±41984.92
12 Dehydrocholic Acid	5982.90±7463.35	3524.03±1624.47	3526.31±4691.29	10743.12±3630.09	25932.87±11667.50	27736.26±13909.90
ω Muricholic Acid	9504.76±715596	8875.91±3049.83	5614.41±7306.10	141400.97±80335.97	263932.58±54767.14	314560.47±64585.18
α/β Muricholic acid	6614.09±6477.68	15970.27±6548.89	12854.28±16443.77	104229.53±25378.65	310467.25±124015.19	446102.24±134109.15
Hyocholic acid	ND	ND	ND	1993.88±1504.40	2402.62±766.86	3325.34±858.03
Cholic acid	10768.04±9751.40	16062.46±6774.92	14513.65±19584.86	4918.26±4337.96	83585.83±82149.69	92569.64±75383.67
Taurochenodeoxycholic Acid	205.31±126.62	1884.39±1866.13	895.85±623.29	1213.77±406.44	3635.21±1601.95	10196.65±6977.81
Taurodeoxycholic Acid	1619.69±1268.99	1745.75±1420.30	1389.52±850.68	994.59±392.36	6086.81±4191.70	13678.11±13731.36
Tauro-ursodeoxycholic Acid	599.47±185.14	902.19±154.69	696.85±394.72	533.87±91.95	2073.17±1341.51	3336.03±2403.10
Taurohyodeoxycholic Acid	ND	ND	ND	423.18±189.83	1154.92±634.43	3067.46±2310.00
Taurocholic Acid	3139.05±1493.79	2748.90±2561.92	1571.36±1345.04	8124.67±1818.68	4898.07±1823.25	10453.98±8743.77
Tauro-ω,α,β Muricholic Acid	3182.83±1101.32	6324.24±4040.42	3319.21±1957.93	8889.43±1268.29	24002.12±15654.38	32360.63±23481.65

Supplementary Table S1: Relative bile acid intensities in the plasma and faeces of BL, HFD and HFD+P mice gathered from UPLC-MS profiling. Data is presented as the mean ± SD from for 4 (plasma) or 6 (faeces) mice in each group. Faecal data is corrected to dry weight of stool.

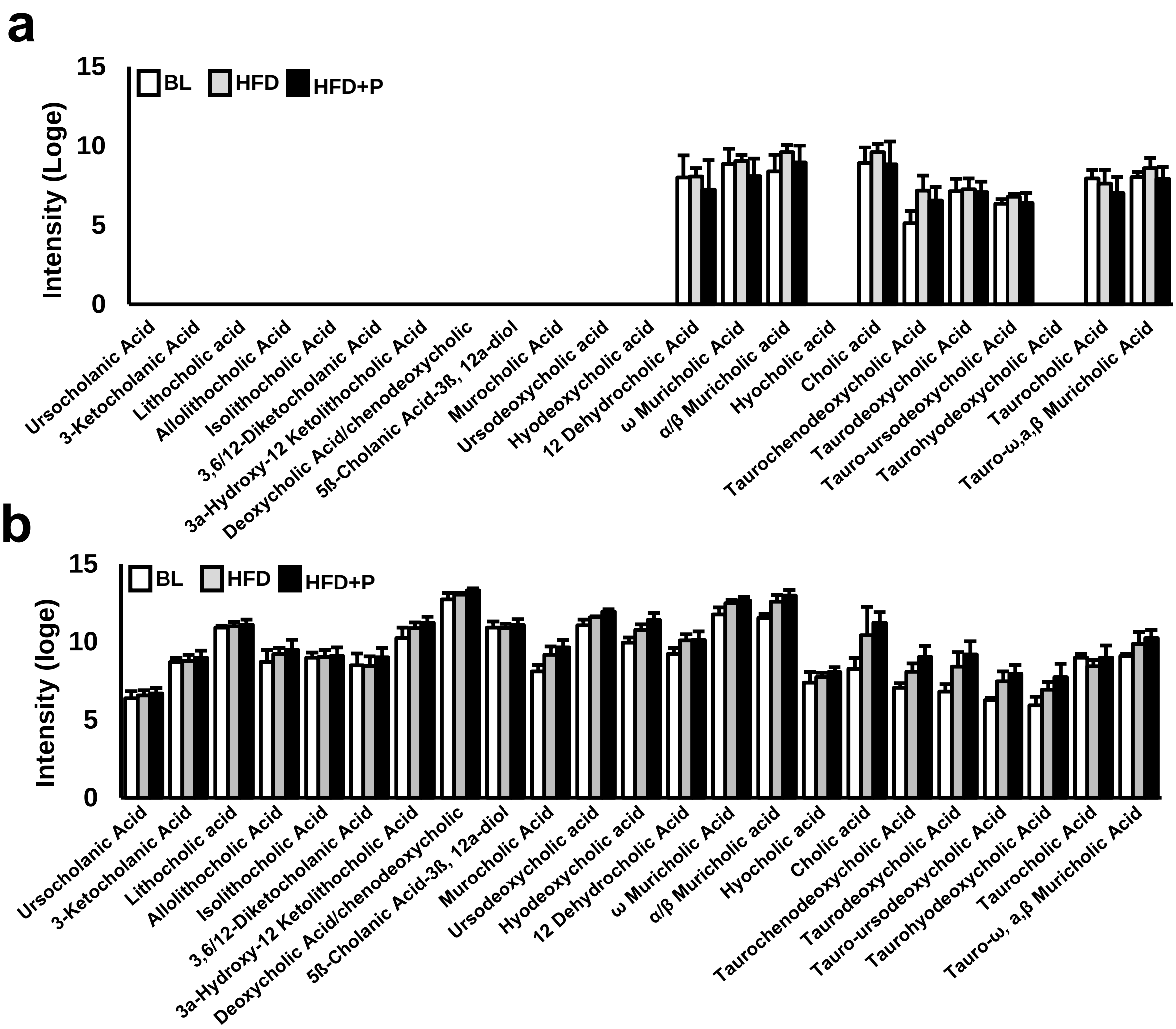

Supplementary Figure S2: BA intensity profiles. Relative intensities of *(a)* plasma and *(b)* faecal bile acid signatures gathered from UPLC-MS analysis are presented for BL, HDF and HFD+P mice. The data is represented as the mean of scaled ($\log_e$) data ± SD for 4 (plasma) or 6 (faeces) mice in each group.

Gene	Forward Primer (5’ to 3’)	Reverse Primer (5’ to 3’)	Ref
mFXR	TCCAGGGTTTCAGACACTGG	GCCGAACGAAGAAACATGG	2
mFGF15	ACGTCCTTGATGGCAATCG	GAGGACCAAAACGAACGAAATT	3
mSHP	CGATCCTCTTCAACCCAGATG	AGGGCTCCAAGACTTCACACA	3
mCYP7A	AACAACCTGCCAGTACTAGATAGC	GTGTAGAGTGAAGTCCTCCTTAGC	4
mNPC1L1	GGCTCCATCTGGAGTAGCTG	ATCGCACTACCATCCAGGAC	5
mABCG-5	CGTGGCGGACCAAATGATT	CCACTGGAAATTCCCCCAAA	6
mABCG-8	GAGCTGCCCGGGATGATA	CGGAAGTCATTGGAAATCTG	7
mABCA-1	TGGAAAACAGTTAATGACCAGCCA	TCCAGTAACAGCTGACATGTTTGT	
mHMGCR	AAGGGTACGGAGAAAGCACT	AATGACGCTTCACAAACCA	8
mβ-Actin	ACACCCGCCACCAGTTCGCCAT	CACACCCTGGTGCCTAGGGCGGCCCACGATG	9

Supplementary Table S2: Oligonucleotide primer sequences

Supplementary reference

1 Gorenjak, M. *et al.* Improvement of lipid profile by probiotic/protective cultures: study in a non-carcinogenic small intestinal cell model. *New Microbiol* **37**, 51-64 (2014).

2 Udayappan, S. *et al.* Oral treatment with Eubacterium hallii improves insulin sensitivity in db/db mice. *Npj Biofilms And Microbiomes* **2**, 16009, doi:10.1038/npjbiofilms.2016.9(2016).

3 Chen, M. L. *et al.* Resveratrol Attenuates Trimethylamine-N-Oxide (TMAO)-Induced Atherosclerosis by Regulating TMAO Synthesis and Bile Acid Metabolism via Remodeling of the Gut Microbiota. *MBio* **7**, e02210-02215, doi:10.1128/mBio.02210-15 (2016).

4 Kong, B. *et al.* Mechanism of tissue-specific farnesoid X receptor in suppressing the expression of genes in bile-acid synthesis in mice. *Hepatology* **56**, 1034-1043, doi:10.1002/hep.25740 (2012).

5 Catry, E. *et al.* Ezetimibe and simvastatin modulate gut microbiota and expression of genes related to cholesterol metabolism. *Life Sci* **132**, 77-84, doi:10.1016/j.lfs.2015.04.004 (2015).

6 Kawase, A., Hata, S., Takagi, M. & Iwaki, M. Pravastatin Modulate Niemann-Pick C1-Like 1 and ATP-Binding Cassette G5 and G8 to Influence Intestinal Cholesterol Absorption. *J Pharm Pharm Sci* **18**, 765-772 (2015).

7 Huang, Y. *et al.* Lactobacillus acidophilus ATCC 4356 prevents atherosclerosis via inhibition of intestinal cholesterol absorption in apolipoprotein E-knockout mice. *Appl Environ Microbiol* **80**, 7496-7504, doi:10.1128/AEM.02926-14 (2014).

8 Song, M. *et al.* Effect of Lactobacillus acidophilus NS1 on plasma cholesterol levels in diet-induced obese mice. *J Dairy Sci* **98**, 1492-1501, doi:10.3168/jds.2014-8586 (2015).

9 Moss, J. W. *et al.* A Unique Combination of Nutritionally Active Ingredients Can Prevent Several Key Processes Associated with Atherosclerosis In Vitro. *PLoS One* **11**, e0151057, doi:10.1371/journal.pone.0151057 (2016).
